# Fed-Batch Production of Bacterial Ghosts Using Dielectric Spectroscopy for Dynamic Process Control

**DOI:** 10.3390/microorganisms4020018

**Published:** 2016-03-24

**Authors:** Andrea Meitz, Patrick Sagmeister, Werner Lubitz, Christoph Herwig, Timo Langemann

**Affiliations:** 1 Research Center Pharmaceutical Engineering (RCPE) GmbH, Inffeldgasse 13, Graz A-8010, Austria; andrea.meitz@tuwien.ac.at (A.M.); mail@timo-langemann.de (T.L.); 2 Research Division Biochemical Engineering, Institute of Chemical Engineering, Vienna University of Technology, Gumpendorfer Strasse 1A 166/4, Vienna A-1060, Austria; patrick.sagmeister@exputec.com; 3 Biotech Innovation Research Development and Consulting (BIRD–C) GmbH & Co KG, Dr.-Bohr-Gasse 2-8, Vienna A-1030, Austria; werner.lubitz@bird-c.at; 4 Center of Molecular Biology, University of Vienna, Dr.-Bohr-Gasse 9, Vienna A-1030, Austria

**Keywords:** E-lysis, bacterial ghosts, process analytical technology (PAT), online biomass monitoring, dielectric spectroscopy, control strategy

## Abstract

The Bacterial Ghost (BG) platform technology evolved from a microbiological expression system incorporating the *ϕX*174 lysis gene *E*. E-lysis generates empty but structurally intact cell envelopes (BGs) from Gram-negative bacteria which have been suggested as candidate vaccines, immunotherapeutic agents or drug delivery vehicles. E-lysis is a highly dynamic and complex biological process that puts exceptional demands towards process understanding and control. The development of a both economic and robust fed-batch production process for BGs required a toolset capable of dealing with rapidly changing concentrations of viable biomass during the E-lysis phase. This challenge was addressed using a transfer function combining dielectric spectroscopy and soft-sensor based biomass estimation for monitoring the rapid decline of viable biomass during the E-lysis phase. The transfer function was implemented to a feed-controller, which followed the permittivity signal closely and was capable of maintaining a constant specific substrate uptake rate during lysis phase. With the described toolset, we were able to increase the yield of BG production processes by a factor of 8–10 when compared to currently used batch procedures reaching lysis efficiencies >98%. This provides elevated potentials for commercial application of the Bacterial Ghost platform technology.

## 1. Introduction

Bacterial Ghosts (BGs) are empty cell envelopes derived from Gram-negative bacteria. Controlled expression of the single cloned bacteriophage *ϕ*X174 lysis gene *E* [1] leads to the fusion of the cytoplasmic (inner) and outer cell membrane (IM/OM, respectively) followed by expulsion of the cytoplasmic content due to the osmotic pressure difference. This process is referred to as protein E-mediated lysis (E-lysis) and forms a membrane-spanning tunnel structure [2,3] in areas of potential cell division [4]. Observations on the E-lysis process showed that E-lysis efficiency strongly depends on the growth phase of the host cell and its autolytic system [5,6,7,8]. It has been shown that stationary phase bacteria do not form BGs even though protein E is accumulating in the IM. When provided with a growth impulse (such as addition of fresh medium), E-lysis is immediate [1]. Production of BGs in a simple batch procedure has been described [9]. The efficacy of BG production has been defined as lysis efficiency (LE, ratio of BGs formed/viable cells before induction of E-lysis) which is based on plate counting [9]. Recently, an advanced method for rapid determination of LE using multi-parameter flow cytometry (FCM) was reported [10].

Dielectric spectroscopy—or permittivity measurements—has emerged as a valuable tool for on-line monitoring and control of mammalian [11,12,13,14], yeast [15,16,17] and microbial fermentation processes [18,19]. The principle of dielectric spectroscopy relies on the polarizability of cell membranes and has been described in detail [20,21,22,23]. Permittivity measurements of a cell suspension are performed at two different frequencies, a high frequency (e.g., 10 MHz) representing the non-cellular background of the system and at a low frequency accounting for viable cells. The choice for the low frequency is dependent on the target cell properties, e.g., size and morphology, and is usually in the range of 1 MHz [24]. The relative permittivity Δ*ε* [pF/cm] of the cell suspension is then defined as the permittivity difference between the two frequencies. The relative permittivity can be correlated to the membrane-enclosed volume of viable cells, and, therefore, in many cases to the concentration of viable biomass in the suspension as only viable cells with intact, polarizable membranes contribute to the measurement [23]. General linearity between biomass concentration and permittivity was shown for bacterial fermentations [18,25], allowing for real-time estimation of the viable biomass concentration. In addition, the term bio-density (biomass/permittivity) has been introduced in case physiological changes (e.g., induction of heterologous protein expression) produce deviation from such linearity [25]. Monitoring of E-lysis by dielectric spectroscopy has recently been reported for technical applications of BGs [26].

Another tool for on-line estimation of viable biomass is a so-called soft-sensor. In contrast to hard-type sensors which measure on-line accessible signals, e.g., turbidity, fluorescence or permittivity [27], a soft-sensor uses signals from hard-type sensors in order to compute unquantifiable process variables, such as the biomass concentration. Different types of soft-sensors have been reviewed [24,28]. Data driven soft-sensors that use statistical regressions derived from multivariate data analysis but need huge training data sets to build process models were successfully applied [29,30]. During bioprocess development, often those training data sets are not available and elemental balancing approaches are the primary choice for the design of a soft sensing strategy. One prominent approach is a first principle soft sensor that estimates viable biomass based on elemental balancing [31,32]. The value of such first principle soft-sensors has been demonstrated [25,33,34]. In contrast to dielectric spectroscopy, which measures the total volume of viable cells, soft-sensors compute the actual biomass from the metabolic activity of the culture [32] and thus also provide capability for dynamic process control [35].

The Food and Drug Administration (FDA) launched an initiative to encourage manufacturers to develop more science and risk based pharmaceutical processes [36]. The prospected benefit is a deeper product and process understanding resulting in a higher operational flexibility. Primary tools for science based process development are risk assessments, design of experiments (DoE) and process analytical technology (PAT) [37,38]. Major steps are the identification of the product’s critical quality attributes and the investigation of the effect of potential critical process parameters on product quality or process performance. Once the mode of action is understood, suitable control strategies can be developed to ensure consistent process performance. PAT can be described as “A system for designing, analyzing, and controlling manufacturing through timely measurements (*i.e.*, during processing) of critical quality and performance attributes of raw and in-process materials and processes with the goal of ensuring final product quality” [39].

The biotechnological product Bacterial Ghosts finds possible applications as candidate vaccines, drug delivery vehicles or in the production of recombinant proteins [9,40,41,42,43,44,45,46,47,48,49,50]. The broad spectrum of applications urges for an economic, robust and scalable production procedure. Current BG production yields ~5 g/L biomass before E-lysis induction (LI) and LEs of 99% [9]. Using fed-batch procedures, the yield of BG production could be extended possibly by the order of one magnitude. However, the highly dynamic and biologically complex process of E-lysis possesses exceptional demands towards process understanding and control. So far, no real-time signal for the estimation of viable biomass during the lysis process is available. A real-time signal describing the cells’ physiological state under dynamic conditions would allow a tailored feeding strategy circumventing excess substrate feeding or critical substrate limitation.

Within this contribution, we want to demonstrate that one can establish a transfer function correlating the permittivity signal with the viable biomass concentration estimated by a soft-sensor during non-induced fed-batch fermentation. This transfer function is then used to estimate the residual viable biomass from the permittivity signal during E-lysis. The subsequent control strategy is discussed as a PAT approach for high-density production of Bacterial Ghosts.

## 2. Methods

### 2.1. Strain

Native *Escherichia coli* Nissle 1917 (DSM 6601, O6:K5:H1) carries two cryptic plasmids (pMUT1, pMUT2) protecting the strain against mobile genetic elements. Removing those plasmids yields *E. coli* Nissle 1917 Δ (EcN) which was provided by Ardeypharm GmbH (Herdecke, Germany). EcN was transformed with plasmid pGLysivb carrying the temperature-inducible gene *E* cassette and a Gentamicin resistance cassette. Lysis gene *E* is under tight control of a modified λ*p_L_*/λ*p_R_*-*c*I857 promoter/operator system that allows for cell growth at 35 °C and expression of the target gene *E* by a rapid temperature up-shift to 42 °C. Plasmid pGLysivb was provided by BIRD-C GmbH & CoKG (Vienna, Austria).

### 2.2. Media

A defined medium as described by DeLisa *et al.* [51] was used, and the concentration of the glucose feed solution (*S*_0_) was 400 g/L. The batch medium was supplemented with gentamicin sulfate (20 mg/L, ROTH, Karlsruhe, Germany) for plasmid retention and 1 g/L polypropylene glycol 2000 (PPG, Sigma, St. Louis, MO, USA) as antifoam agent. The pH of the medium was adjusted to 7.2 before inoculation by addition 5 M KOH.

### 2.3. Plasmid Stability

Master and working cell banks of EcN were streaked on standard agar plates in order to obtain single colonies. From each cell bank, 200 colonies were picked and applied fifty-fifty on plates with and without gentamicin. The ratio of colonies from the plates with gentamicin (C_+Gent_) divided by the number of colonies from plates without gentamicin (C_−Gent_) gives the plasmid stability in percent as given in Equation (1).

(1) $$\text{Plasmid stability}=\left(\frac{C_{+Gent}}{C_{-Gent}}\right)\cdot 100\%$$

Equation (1): Determining the plasmid stability.

### 2.4. Bioreactors

Pilot scale fed-batch experiments were carried out in a Techfors-S (Infors-HT, Bottmingen, Switzerland) fully automated stainless steel bioreactors (20 L working volume). The pH was controlled at pH 7.2 by addition of 6 M NH_4_OH (base) and 1 M H_3_PO_4_ (acid). Inlet air and oxygen flows were controlled using suitable red-y mass flow controllers (MFC; GSC-C9SA-BB12/air, GSC-C3SA-BB26/oxygen; Vögtlin, Aesch, Switzerland). The culture vessel was sterilized *in situ* at 121 °C for 20 min prior to inoculation.

For the screening-scale experiments, a DASGIP^®^ (Jülich, Germany) Parallel Bioreactor System for Microbiology with four glass vessels (2.7 L working volume) and equivalent instrumentation (TC4SC4, PH4PO4, MX4/4, MP8) was used. The prepared glass vessels were autoclaved at 121 °C for 20 min prior to inoculation.

### 2.5. Fermentation

All fed-batch processes for BG production were divided into three phases: (a) batch phase for initial biomass formation; (b) fed-batch phase for biomass propagation and (c) induction phase for E-lysis with continued feeding at elevated temperatures. Batch fermentations were inoculated from a pre-culture (OD_600_ 1–2). At the end of the batch phase (as detected by a sudden decline in the CO_2_ concentration in the off-gas), the exponential fed-batch phase (b) was started by implementing a defined specific substrate uptake rate *q_S_* which was governed by controlling the feed rate *F* (see equations below). Deviations between the feed rate set-point and the actual value (as determined from the decreasing weight of the feed container over time) were fed to a PID controller that actuated the feed pump set-point in order to maintain the desired specific substrate uptake rate. All relevant process parameters for batch (a) and fed-batch phase (b) are given in Table 1.

The feed rate set-point was calculated based on the differential equations describing a substrate-limited fed-batch situation (Equations (2) and (3)):

(2) $$\frac{dX}{dt}\cdot V+\frac{dV}{dt}\cdot X=\mu \cdot X\cdot V$$

Equation (2): Mass balance for biomass (*X*) formation with changing volume (*V*). *µ*: specific growth rate.

(3) $$q_{S}\cdot X=-\frac{1}{Y_{X/S}}\cdot \frac{dX}{dt}$$

Equation (3): Mass balance for substrate (*S*) consumption; *q_S_*: specific substrate uptake rate; *Y_X/S_*: biomass yield coefficient.

Integrating and rearranging gives the initial feed rate *F*_0_ and the subsequent feeding regime *F*(*t*) as depicted in Equations (4) and (5), respectively.

(4) $$F_{0}=\frac{\mu \cdot X_{0}\cdot V_{0}}{Y_{X/S}\cdot S_{0}}\cdot \rho_{f}=\frac{q_{S}\cdot X_{0}\cdot V_{0}}{S_{0}}\cdot \rho_{f}$$

Equation (4): Calculation of the initial feed rate *F*_0_ for the fed-batch phase in [g/h]. *X*_0_: initial biomass concentration (end of batch phase); *V*_0_: initial (batch) volume; *S*_0_ substrate (glucose) concentration in the feed; *ρ _f_*: density of the feed.

(5) $$F\left(t\right)=F_{0}\cdot e^{\mu \cdot t}$$

Equation (5): Calculation of the feed rate *F(t)* in the fed-batch phase (b) in [g/h].

### 2.6. Off-Gas Analytics

The off-gas concentrations of the Techfors-S fermentations (CO_2_ and O_2_) were quantified using a gas analyzer (Dr. Marino Müller AG, Egg, Switzerland) using infrared and paramagnetic principle, respectively. Off-gas analysis for the DASGIP quad system was done using a GA4 module (DASGIP, Jülich, Germany) featuring BlueSens sensors for O_2_ and CO_2_ (BlueSens, Herten, Germany).

### 2.7. Dielectric Spectroscopy

A Biomass 220 system with a 25 mm annular type probe (ABER Instruments, Aberystwyth, Wales, UK) was applied for in-line measurements of the relative permittivity Δε, which was estimated at frequencies of 10 MHz accounting for non-cellular background and 1.0 MHz attributed to living bacteria.

### 2.8. Process Management and Soft-Sensor

The process control system (PCS) Lucullus (SecureCell AG, Schlieren, Switzerland) was used for monitoring and control of the pilot-scale processes. A tool for estimation of the current broth volume was implemented in Lucullus-integrated interface tool Sim-Fit. A strategy for controlling *q_S_* during E-lysis phase was implemented using simple calculator devices as outlined in the results section. Screening experiments were monitored and controlled using the software DASGIP Control V.4.5 (DASGIP, Jülich, Germany).

A first principle rate-based soft-sensor was used for the real-time estimation of viable biomass during the fed-batch phase using a cumulative calculation approach. The approach is based on the real-time calculation of metabolic reaction rates. Generation of an over-determined equation system using the Degree of Reduction (DoR) and the carbon balance allows for estimating unknown rates. The fermentation volume can be calculated through a mass balance. The input term considers mass input through substrate and base inflow as well as oxygen fixation by the microorganisms. The output term considers water stripping, sampling and mass loss through carbon dioxide production. A detailed description of this approach was published [35].

### 2.9. Biomass Concentrations

Biomass concentrations as dry cell weight (DCW) were determined off-line for validation of the soft-sensor. The DCW was quantified gravimetrically after drying for a minimum of 72 h at 105 °C. Samples were centrifuged (5000 rpm, 10 min), and the pellet was washed with distilled water.

### 2.10. Flow Cytometry

For flow cytometry (FCM), a Cube 6 system (Partec, Münster, Germany) with a 488 nm blue solid state laser was used. Samples were diluted appropriately and stained with two fluorescent dyes: RH 414 (abs./em.: 532/718 nm, final conc.: 3 nM) for staining cell membranes and DiBAC_4_(3) (abs./em.: 493/516 nm, final conc.: 0.75 nM) for determining cell viability (both dyes: AnaSpec, Fremont, CA, USA). RH 414 signals were picked up in channel FL2 (orange) defining a gate G1 for exclusion of non-cellular background. Combination of FSC and FL1 (green) signals were used to identify regions for populations of living bacteria (R1), dead but intact bacteria (R2) and Bacterial Ghosts (R3). The method as well as definition of regions R1–R3 have been described in detail [10].

### 2.11. E-Lysis Efficiency

All given values for E-lysis efficiency (LE) are based on FCM data. LE at any time *t* after LI can be calculated directly as the ratio of cell counts (CC) in the region R3 (BGs) and total cell counts as given in Equation (6).

(6) $$\text{LE}=\left(\frac{{\text{CC}}_{R3}\left(t\right)}{\sum_{i}{\text{CC}}_{Ri}\left(t\right)}\right)\cdot 100\%$$

Equation (6): Calculating the E-lysis efficiency from FCM data.

## 3. Results

The strain under investigation, *i.e.*, EcN (pGLysivb), was characterized in prior studies [52] where biomass formation was correlated with discrete measurements of substrate (*i.e.*, glucose) consumption and by-product (*i.e*., acetate) formation. During unlimited batch growth on defined medium (35 °C, C-source: 20 g/L glucose) EcN shows a maximum specific growth rate of *µ_max_* = 0.75 h^−1^ and a maximum specific substrate uptake rate *q_S,max_* = 1.67 g/gh. The biomass yield coefficient for such conditions is *Y_X/S_* = 0.45 g/g while ~1 g/L acetate is produced towards the end of batch phase. In substrate-limited (*i.e.*, C-limited) fed-batch conditions with specific substrate uptake rates distinctly lower than *q_S,max_* (here: *q_S_* < 1.1 g/gh), the strain shows no acetate formation and the biomass yield increases to *Y_X/S_* = 0.5 g/g.

### 3.1. Identification of Critical Process Parameters

**Figure 1:**
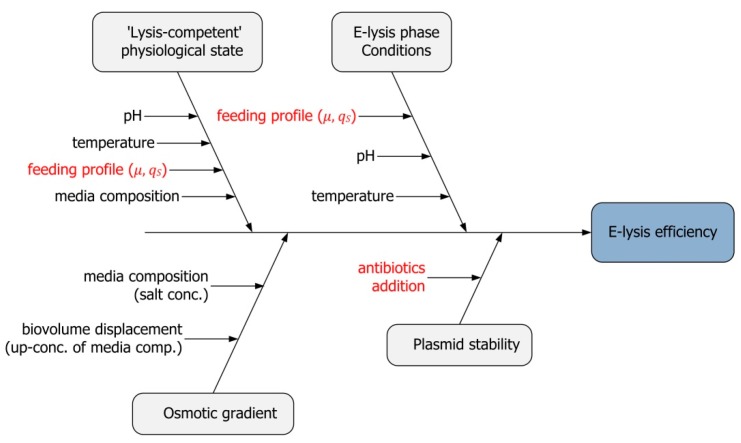
Ishikawa diagram depicting the potential critical process parameters as a result of a risk assessment. The critical process parameters potentially affecting the critical quality attribute of lysis efficiency are marked in red and were selected for further studies.

To follow a science-based approach, all critical parameters having potential negative effects on the overall lysis efficiency (LE) had to be identified, both during fed-batch phase (b) as well as during E-lysis phase (c). This was done in a classical risk assessment using a cause-and-effect (Ishikawa) diagram with the focus on LE as a critical quality attribute. All potential critical process parameters are included and rated due to their potential criticality, as shown in Figure 1. Parameters of high criticality are marked in red in the diagram and were selected for further characterization studies.

The feeding profile was rated the major critical parameter as fed-batch processes are conducted in a state of substrate limitation. For both fed-batch phase (b) and E-lysis phase (c), a potential negative effect of low growth rates on E-lysis performance through the formation of a stationary, non-dividing subpopulation must be prevented.

Plasmid stability as second critical parameter for LE was investigated as described above. The E-lysis plasmid (pGLysivb) was retained in 100% of the population when grown on Agar plates without addition of antibiotics.

### 3.2. Effect of Specific Substrate Uptake Rate during Fed-Batch Phase on Lysis Efficiency

In order to investigate the influence of growth conditions during fed-batch phase (b), screening experiments with different feeding profiles (specific substrate uptake rate *q_S_* *_(b)_* = 0.4, 0.7 and 1 g/gh) during fed-batch phase (b) were conducted. Screening-scale experiments were done in parallel, aiming for a biomass concentration of 15 g/L at the end of the fed-batch phase (b). The feed flow rate was set to a fixed value corresponding to a specific substrate rate *q_S_* *_(c)_* = 1.0 g/gh at the beginning of the E-lysis phase (c) and kept constant all during the lysis phase. LE was determined via FCM at-line measurements as described above. The critical quality attribute LE as a function of the specific substrate uptake rate *q_S_* *_(b)_* during fed-batch phase (b) is shown in Table 2.

It can be concluded that, in the investigated range, the specific substrate uptake rate *q_S__(b)_* during fed-batch phase (b) has no impact on the lysis competence of the cells, which gives freedom to operate within this range. Process design was therefore continued with a fixed *q_S__(b)_* = 0.6 g/gh, after considering economic and process technological aspects such as e.g., process time and oxygen transfer limitations. 

### 3.3. Determination of E-lysis Conditions Using Dielectric Spectroscopy

An experimental setup to investigate the conditions for lysis onset was established as follows. For this experimental approach, the strain under investigation was grown at 20 L scale to a biomass concentration of X = 30 g/L in an exponential fed-batch (*q_S_* *_(b)_* = 0.6 g/gh, T = 35 °C). Expression of lysis gene *E* was induced by shifting the temperature to 42 °C while *q_S_* *_(c)_* was reduced to 0.1 g/gh (based on the current biomass concentration) and kept on this low level for one hour in order to accumulate protein E but prevent E-lysis. The feed flow rate was then linearly increased within a time frame of two hours. The actual viable biomass concentration was followed by permittivity measurements and a sudden and steep signal drop caused by disruption of the membrane potential [6] was expected at the point of E-lysis onset.

**Figure 2:**
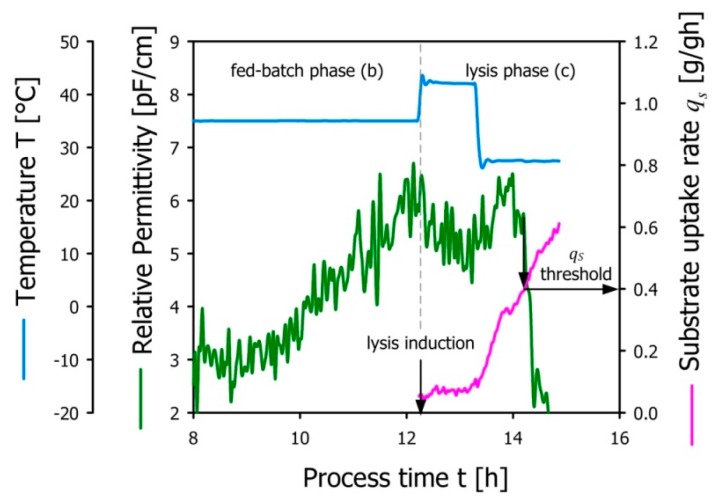
Determination of E-lysis onset based on permittivity measurements (green line) by increasing the specific substrate uptake rate *q_S_* (pink line). The threshold value of E-lysis onset was found for *q_S_* = 0.4 g/gh.

Figure 2 shows the course of the experiment beginning with the fed-batch phase (b). The values for *q_S__(c)_* were calculated in retrospect from the feed flow rate based on the assumption that the change in biomass concentration was negligible in the early stage of phase (c). This assumption is supported by the fact that the permittivity signal stagnates after LI before a significant drop indicated the onset of E-lysis at ~14.25 h process time. From that, we concluded that the minimum value for *q_S__(c)_* in order to see the first indicators for E-lysis is approximately 0.4 g/gh. Since *q_S__(c)_* is calculated as a function of the feed flow rate and the biomass concentration at the end of the fed-batch phase (b), the values depicted in Figure 2 after E-lysis onset represent *q_S__(c)_* based on the feed-forward approach as described above.

### 3.4. Demonstration of the Developed Permittivity Controlled Feeding Strategy

We have developed a control strategy aiming to combine the real-time signal for viable biomass estimation provided by a soft-sensor with the permittivity signal that further allows following dynamic process conditions like E-lysis upon membrane depolarization.

**Figure 3:**
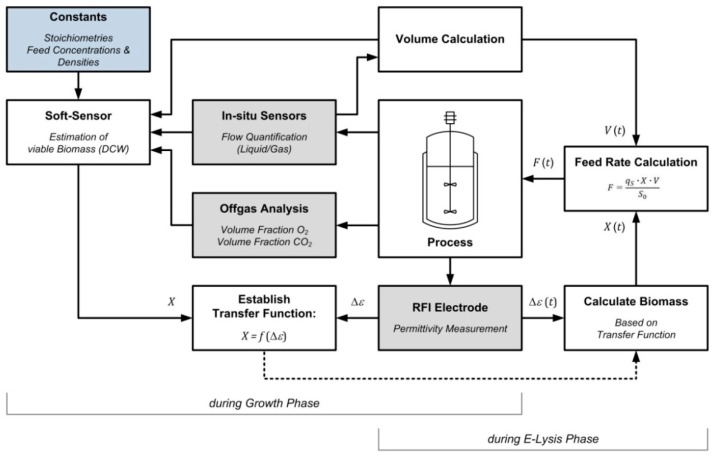
Schematic drawing of the feed control strategy during the E-lysis phase. Grey boxes indicate measurements, white boxes indicate calculation, blue boxes provide known constants.

Figure 3 shows a schematic drawing of the suggested control strategy. During fed-batch phase (b), the biomass concentration is estimated by a soft-sensor, while, at the same time, the bio-volume of viable bacteria is measured through dielectric spectroscopy. Towards the end of phase (b), a transfer function is established that allows estimation of the viable biomass X as a function of the relative permittivity Δε. At the beginning of E-lysis phase (c), this transfer function is implemented in the PCS where calculator tools estimate the viable biomass X(t) as well as the current volume V(t).

Consequently, the loss of membrane potential can be followed as a drop in the permittivity signal. With the above mentioned transfer function, the PCS is then able to adjust the feed rate *F(t)* proportionally to the decrease of viable biomass, thereby maintaining a constant specific substrate uptake rate *q_S_* *_(c)_* during E-lysis phase (c).

**Figure 4:**
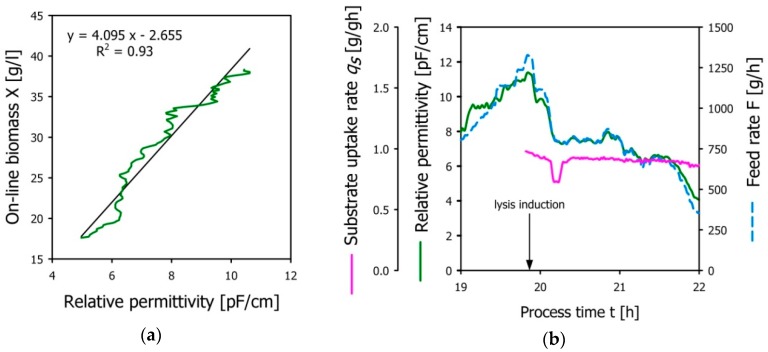
(**a**) Correlation of in-line permittivity measurements with on-line biomass data (transfer function); (**b**) Effective permittivity based feed-rate control. The actual feed-rate (dashed blue line) follows the drop of the permittivity signal (green line) during the E-lysis process and provides a constant specific substrate uptake (pink line).

A fermentation run with EcN (pGLysivb) was conducted to demonstrate the applicability of the capacitance-controlled feeding strategy using the 20 L bioreactor setup as described above. The permittivity signal recorded during fed-batch phase was correlated with the output of the soft-sensor in the fed-batch phase establishing the transfer function *X = f* (Δ*ε*) as mentioned above. Figure 4a shows the correlation of online biomass data from the soft-sensor with the in-line permittivity signal (b(0) = −2.564, b(1) = 4.094, *R*^2^ = 0.93). At the point of lysis induction (indicated by the arrow in Figure 4b, the feed-forward controller for the exponential flow rate was turned off and the permittivity-based feed rate control was implemented. Figure 4b shows that the feed rate implemented by the permittivity-driven control strategy closely follows the course of the permittivity and also that the specific substrate rate *q_S__(c)_* could be maintained at a constant average level of 0.92 g/gh over the whole E-lysis phase (c).

**Figure 5:**
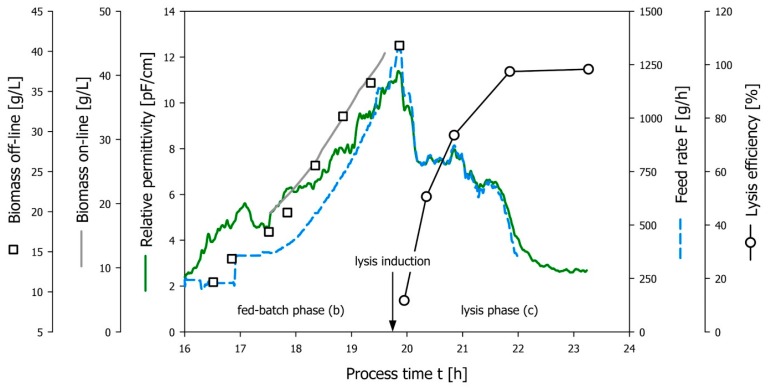
Demonstration of the described control method during the E-lysis phase. The feed-rate follows the permittivity signal nicely after lysis induction. The E-lysis efficiency (based on FCM data) increases over the duration of the lysis phase and reaches a value > 98%.

Figure 5 summarizes the relevant process phases to demonstrate the successful implementation of the permittivity based feed-rate control for the production of BGs. The batch phase yielded a biomass concentration X = 8.3 g/L at which a feed-forward fed-batch was started by setting the feeding profile to a target specific substrate uptake rate *q_S_* *_(b)_* = 0.6 g/gh. Biomass propagation is followed by dielectric spectroscopy measuring the permittivity, offline biomass sampling and a soft-sensor predicting the viable biomass as described above. Offline biomass samples were taken at thirty-minute intervals to retrospectively validate the biomass estimation by the soft-sensor. Good correlation between online and offline biomass measures as well as linear proportionality between permittivity signal and viable biomass (see also Figure 4b) was demonstrated during the fed-batch phase. Following the accumulation of 40 g/L biomass, the temperature was increased to 42 °C to open the temperature inducible promoter for E-lysis, and the permittivity based feed-rate control was started. The sudden drop in permittivity indicating the loss of membrane potential due to E-lysis is in good agreement with the at-line FCM data, showing increasing lysis efficiency with a final LE = 98.3% at the end of E-lysis phase (c).

## 4. Discussion

Literature has so far described E-lysis of Gram-negative bacteria in a state of unlimited exponential growth, usually in some sort of batch-like approach [9,43]. It was shown that E-lysis is dependent on a certain level of physiological activity as well as an intact membrane potential [53], whereas growth stagnation before lysis induction resulted in lysis-incompetent populations [1]. Therefore, one of the major constraints for successful E-lysis is that, until the time-point of lysis induction, the bacteria are maintained in lysis-competent conditions, *i.e*., that the population is in a physiological state that allows every bacterium to go through the cycle of cell division [1].

In fed-batch processes, growth conditions are specified by the feeding profile defining a constant substrate uptake rate that is usually chosen at the lower end of the physiologically possible range. However, such low substrate uptake rates translate into low specific growth rates that presumably mimic conditions impeding E-lysis onset. Consequently, one expects that minimum substrate uptake rates *q_S_* must be maintained during fed-batch (b) and E-lysis phase (c), the latter of which can be regarded a threshold for E-lysis onset. Development of a fed-batch production process for BGs therefore involves investigating the effects of the growth conditions on the overall LE by adjusting the specific substrate uptake rate *q_S_*—here considered as a biomass specific measure of total metabolism—during fed-batch (*q_S__(b)_*) and E-lysis phase (*q_S__(c)_*). We have shown that the specific substrate rate *q_S__(b)_* during fed-batch phase in a range of 0.4 to 1.0 does not influence E-lysis performance. For the specific substrate rate during lysis phase, however, we found a threshold value of 0.4 g/gh below which no signs of E-lysis were observed. From these findings, we conclude that at specific substrate uptake rates < 0.4 g/gh, which corresponds to specific growth rates of *µ* < 0.2 h^−1^, the vast majority of the bacterial population converges into a state similar to the stationary phase where E-lysis is inhibited. These observations align well with the abovementioned effects of low physiological activity on E-lysis performance.

The gained process knowledge now demanded for a control strategy that is capable of maintaining the conditions required for E-lysis. Feeding sufficient amounts of substrate during E-lysis phase (c) was equally important to not overfeeding the culture in order to avoid accumulation of substrate. The fed-batch feeding concept as described above (Equations (2) and (3)) comprises a couple of constants as well as the current fermentation volume and viable biomass concentration. The E-lysis phase (c) is a highly dynamic phase where the number of viable cells decreases rapidly and exponentially, *i.e.*, the change in viable biomass predominates the changes in volume. In order to control the specific substrate uptake rate *q_S__(c)_* during this phase, reliable on-line estimation of the viable cell concentration was crucial. The applied soft-sensor delivered robust and accurate estimations of the viable biomass concentration for standard growth conditions during fed-batch phase (b) but failed to reflect predicting the viable biomass concentration during dynamic, undefined process conditions.

Literature reports that E-lysis results in a sudden loss of membrane polarizability [6]. We could show that this effect is immediately observable in permittivity measurements of the fermentation broth as cells affected by E-lysis no longer contribute to the cumulated permittivity of the culture. Consequently, the progress of E-lysis can be followed directly and quantitatively by dielectric spectroscopy measurements in real time. Applying the above described transfer function, the viability information gained from dielectric spectroscopy was translated into the required parameter viable biomass concentration. The PCS was then able to control the feed rate *F(t)* proportionally to the decrease of viable biomass. Using this novel control strategy, we were able to maintain the specific substrate uptake rate *q_S__(c)_* at an average of 92% of the set-point (1.0 g/gh) over the whole lysis phase (c).

With the approach we have presented in this contribution, we were able to produce the microbial product Bacterial Ghosts with a lysis efficiency of LE > 98%, maintaining full control over all process phases. The described fed-batch procedure gives 8 to 10-fold higher yields (BG per liter fermentation broth) compared to the previously described batch process [9] making BG applications more attractive for commercialization. The control approach we have used proved to be an efficient on-line process analyzer, while, at the same time, providing control during manufacturing. This is in accordance with the FDA’s requirements for PAT to accommodate the ability and reliability to measure critical attributes and the achievement of process end points to ensure consistent quality of the output materials and the final product [39].

## 5. Conclusions

- The biological process of E-lysis depends on a certain level of physiological activity; using the specific substrate uptake rate as a biomass specific measure of total metabolism, we were able to determine a threshold value *q_S_* > 0.4 g/gh required for successful E-lysis under substrate-limited conditions.
- We could show that quantifying the relative permittivity of the culture broth as a measure for membrane polarizability, one can follow the drop of viability in a culture affected by E-lysis.
- Viability information gained from permittivity measurements (Δ*ε*) could be aligned with soft-sensor-derived estimation of viable biomass concentrations (*X*) during bacterial growth.
- Using a transfer function *X* = *f* (Δ*ε*), we were able to control the specific substrate uptake rate *q_S_* throughout the entire lysis phase.
- The described fed-batch process increased the productivity of Bacterial Ghost production by a factor of 8–10 reaching lysis efficiencies of >98%.

## Figures and Tables

**Table 1:** Cultivation parameters for batch (a) and fed-batch phase (b).

	Process Parameter	Value
General	growth temperature [°C]	35
	pressure [bar]	1.2/- *
	air flow rate [L/min]	1 vvm
	stirring speed [rpm]	1200/1500 *
Batch (a)	glucose conc. (*S*) [g/L]	20.0
	final biomass *X*_0_ [g/L]	9.0
	volume *V*_0_ [L]	7.0/1.0 *
Fed–Batch (b)	specific substrate uptake rate *q_S_* [g/gh]	0.6/varying *
	oxygen flow rate [L/min]	max. 0.1/1.0 vvm *
	glucose conc. *S*_0_ feed [g/L]	400
	target biomass concentration *X* [g/L]	40

* Deviating values for pilot-scale/screening-scale fermentations.

**Table 2:** Fermentation runs performed with different feeding profiles *qS _(b)_* during fed-batch phase. Varying the feed-profile during fed-batch phase showed no impact on lysis efficiency (LE) as determined by flow cytometry according to Equation (4).

*q_S_* *_(b)_* [g/gh]	R1 [counts/mL]	R2 [counts/mL]	R3 [counts/mL]	LE [%]
0.4	1.04 × 10^9^	*n.d.*	2.20 × 10^10^	95.5
0.7	2.53 × 10^9^	1.20 × 10^6^	2.82 × 10^10^	91.7
0.7	1.30 × 10^9^	5.20 × 10^6^	2.25 × 10^10^	94.5
1.0	3.82 × 10^8^	*n.d.*	1.13 × 10^10^	96.7

*n.d.*, none detected.

## Abbreviations

The following abbreviations are used in this manuscript:

*µ*	specific growth rate
*µ*_max_	maximum specific growth rate
Δε	relative permittivity
BGs	Bacterial Ghosts
DCW	dry cell weight
DoE	design of experiments
DoR	Degree of Reduction
EcN	*Escherichia coli* Nissle 1917
E-lysis	protein E-mediated lysis
F	feed flow rate
FCM	flow cytometry
FDA	Food and Drug Administration
LE	lysis efficiency
LI	E-lysis induction
MFC	mass flow controller
PAT	process analytical technology
PCS	process control system
PID controller	proportional-integral-derivative controller
*q*_S_	specific substrate uptake rate
*q_S,max_*	maximum specific substrate uptake rate
V	(fermentation) volume
X	viable biomass concentration
Y_X/S_	biomass yield coefficient
